# Principal component analysis reveals gender-specific predictors of cardiometabolic risk in 6th graders

**DOI:** 10.1186/1475-2840-11-146

**Published:** 2012-11-28

**Authors:** Mark D Peterson, Dongmei Liu, Heidi B IglayReger, William A Saltarelli, Paul S Visich, Paul M Gordon

**Affiliations:** 1 Laboratory for Physical Activity and Exercise Intervention Research, Department of Physical Medicine and Rehabilitation, University of Michigan, Ann Arbor, MI, USA; 2 Human Performance Laboratory, Central Michigan University, Mt. Pleasant, MI, USA; 3 Exercise and Sport Performance Department, University of New England, Portland, ME, USA

**Keywords:** Pediatrics, Principal component analysis, Cardiorespiratory fitness, Obesity

## Abstract

**Background:**

The purpose of this study was to determine the sex-specific pattern of pediatric cardiometabolic risk with principal component analysis, using several biological, behavioral and parental variables in a large cohort (n = 2866) of 6th grade students.

**Methods:**

Cardiometabolic risk components included waist circumference, fasting glucose, blood pressure, plasma triglycerides levels and HDL-cholesterol. Principal components analysis was used to determine the pattern of risk clustering and to derive a continuous aggregate score (MetScore). Stratified risk components and MetScore were analyzed for association with age, body mass index (BMI), cardiorespiratory fitness (CRF), physical activity (PA), and parental factors.

**Results:**

In both boys and girls, BMI and CRF were associated with multiple risk components, and overall MetScore. Maternal smoking was associated with multiple risk components in girls and boys, as well as MetScore in boys, even after controlling for children’s BMI. Paternal family history of early cardiovascular disease (CVD) and parental age were associated with increased blood pressure and MetScore for girls. Children’s PA levels, maternal history of early CVD, and paternal BMI were also indicative for various risk components, but not MetScore.

**Conclusions:**

Several biological and behavioral factors were independently associated with children’s cardiometabolic disease risk, and thus represent a unique gender-specific risk profile. These data serve to bolster the independent contribution of CRF, PA, and family-oriented healthy lifestyles for improving children’s health.

## Background

The prevalence of obesity has increased significantly and is a primary cause of cardiometabolic comorbidities [1]. Evidence from the National Health and Nutrition Examination Survey (NHANES) [2] has indicated that approximately two-thirds of adolescents present with one or more risk factors, and that nearly 10% of this population has clustering of three or more cardiometabolic abnormalities [3]. Moreover, obesity and insulin resistance (IR) during childhood are demonstrated to track into adulthood [4-6], and appear to be antecedents to adult-onset type 2 diabetes [7] and cardiovascular complications [8]. Evidence further suggests that risk for cardiovascular disease (CVD) originates during childhood [9], and that several risk factors such as obesity, glucose intolerance, and hypertension are associated with premature mortality [10]. However, the clinical value of defining and identifying pediatric patients with the metabolic syndrome (MetS) has been heavily debated, due largely to the instability of a dichotomized diagnosis [11].

Findings also reveal that MetS in childhood is a robust predictor of adult cardiovascular disease [8], and thus early prevention efforts are crucial among pediatric populations. However, adequate guidelines are at present lacking to delineate the structure and norms for risk factor stratification in pediatrics [11]. Barriers to a universally accepted definition of MetS include the fact that clinical end-points emerge infrequently at an early age, and that there is substantial variability in the established normal values across different ages and races [11]. Until recently [12], virtually no research has delineated the sex-specific patterns of cardiometabolic risk among adolescents. We have previously shown that sexual-dimorphic differences indeed have a genetic underpinning [13] that likely also impacts cardiometabolic risk beginning in early phases of life. As a result, the use of adult criteria to classify children and adolescent patients with cardiometabolic abnormalities may significantly misrepresent underlying risk in boys and girls.

Although previous studies have incorporated continuous MetS scores to improve robustness of cardiometabolic risk assessment [14], the respective “weight” of each component on absolute cardiometabolic risk is not accounted for, and thus limits the value of creating a MetScore for risk determination [15]. A more robust method of principal components analysis (PCA) has been proposed [16] that accounts for the loading coefficient of each variable. Although the loading coefficients are limited to the specific data set from which they are derived, this strategy enables the aggregation of key MetS risk components that are known to cluster, and also provides a weighted, continuous MetS risk outcome that is demonstrated to remain stable into young adulthood [17]. PCA has been recently used in pediatric cohorts to aid in the interpretation of statistical MetS clustering [18,19] and subsequent reduction into orthogonal factors. However, the technique has never been incorporated for the purpose of identifying independent, biological and behavioral cardiometabolic risk predictors specific to boys and girls. Indeed, using a sex-stratified analysis will provide a better indication of the unique pattern of cardiometabolic component clustering, which may further identify at-risk pediatrics as well as inform tailored approaches to prevent the establishment of cardiometabolic comorbidities in adulthood. Therefore, the purpose of this investigation was to assess the influence of biological, behavioral and parental factors on sex-specific pediatric cardiometabolic risk, using a PCA-derived continuous MetScore.

## Materials and methods

### Study overview

The Cardiovascular Health Intervention Program (CHIP) is a population-based study of sixth graders that includes a screening component and health education program. Details of the program are previously described in detail [20]. Briefly, screenings include the following cardiometabolic risk factor assessments: physical activity (PA), cardiorespiratory fitness (CRF), body composition, blood pressure, family history, fasted blood lipids, fasted glucose, grip strength, and a blood spot sample for genotyping. Of the data collected, we incorporated traditional MetS components (waist circumference, systolic blood pressure, triglycerides, etc.) for aggregating the outcome of cardiometabolic risk, and numerous predictor variables (cardiorespiratory fitness, BMI, family history, parental smoking, etc.) based on preliminary bivariate analyses and previous/current literature. Equal recruitment of boys (51%) and girls (49%) into the program permits a formal assessment of sex specific characteristics in cardiometabolic risk.

### Participants

Between 2005 and 2008, 6th grade students from 17 mid-area Michigan schools were recruited to participate in CHIP. Among the 4159 children who completed assent and consent forms, 3970 children underwent a complete health risk assessment. The present study was based on data from 3028 children who met all inclusion criteria. The following participants were excluded from the study: (1) 87 children reported to have heart problems; (2) 14 reported to have diabetes; (3) 2 reported to have both heart problem and diabetes; (4) 10 had fasting blood glucose levels >126 mg/dl; and (5) 39 had incomplete measures on MetS components. The resulting n = 2866 students (51.27% female, 95% Caucasian, 10 to 13 years of age) were included in the analysis. The CHIP protocol was reviewed and approved by the institutional review board at Central Michigan University, as well as the administration of each participating school.

### Anthropometric measures

Height was measured to the nearest 0.5 cm using a stadiometer. Body mass was recorded to the nearest 0.5 kg using an electronic scale (BWB-800-Tanita, Toyko Japan). Waist circumference (WC) was measured with a Gulick tape measure at a level midway between the lowest rib and the iliac crest. Body mass index (BMI) was calculated (kg·m^-2^), and obesity prevalence was estimated based on growth chart percentiles developed by the Centers for Disease Control (CDC). A BMI ≥ 85th and < the 95th percentile of age and sex specific reference population [21] was designated as overweight, and BMI ≥ 95th percentile was considered to be obese.

### Blood pressure and serum cardiometabolic parameters

Systolic (SBP) and diastolic blood pressure (DBP) were measured using a sphygmomanometer according to the standard protocol for adolescents. The mean of the two measurements was calculated and used in the analysis. Fasting blood was obtained to measure a lipid profile and glucose levels. Children and parents were instructed to have the child fast for a minimum of 8 h the night before the screening, and refrain from eating breakfast the morning of the screening. Blood samples were analyzed using a calibrated Cholestech LDX cholesterol analyzer (Cholestech Corporation, Hayward CA). A complete glucose/lipid panel measure included HDL-cholesterol (HDL), LDL cholesterol (LDL), non-HDL cholesterol (nHDL), total cholesterol (TC) and triglycerides (TG) and blood glucose.

### Cardiorespiratory Fitness (CRF)

A 7-min step-test (modified Canadian Home fitness test) was used to evaluate each child’s cardiorespiratory fitness level. Using a Polar heart rate monitor model 60911 (Polar Electro, Woodbury, NY), participants performed continuous stepping on a 20-cm set of steps for 7-min. A validated regression equation was used to estimate maximal oxygen consumption (VO_2_) [22]. Cut points for a healthy level of aerobic capacity were ≥ 39 and 42 ml/kg/min for girls and boys, respectively. The validity of this test was confirmed by conducting a treadmill VO_2_ max test among a subset of children (n = 20) (interclass correlation coefficient, *r* = 0.80, p < 0.01).

### Habitual Physical Activity (PA)

PA was assessed by questionnaire during the screening. The questionnaire was developed and validated by the CDC for the Youth Risk Behavior Surveillance System (YRBSS), and has been used to measure progress towards achieving national health objectives [23]. Each student was interviewed about the number of days he/she was physically active during the previous 7-days. Being physically active was defined according to CDC recommendations as accumulating a minimum of 60 min per day for ≥5 days per week, in any kind of PA that raised heart rate and breathing rate above the resting state. Children were categorized as either physically active or not active, based on whether the student was physically active for ≥5 days or not. Children who were not physically active were further divided into two sub-groups: i.e. physically inactive if they were active for <3 days, and moderately-active if they were active for ≥3 but <5 days per week.

### Continuous MetS risk score (MetScore)

The MetScore was calculated according to the method described by Wijndaele et al. [16]. Five MetS components (WC, SBP, TG, HDL and glucose) were analyzed with principal components analysis (PCA). Triglycerides levels were log-transformed to correct for skewed distribution. HDL was multiplied by −1 because it is reciprocally associated with cardiometabolic risk. Only SBP was used in the MetScore because SBP and DBP were highly correlated (see Additional file 1: Table S1). Children with valid measurements in all five risk factors (n = 1482 girls and 1384 boys) were included in the PCA. PCA with varimax rotation was applied to the individual risk factors to derive components that represent large fractions of MetS variance.

### Parental factors and family history

A health history questionnaire was completed by parents or guardian, to determine family history of early CVD and childhood diabetes. Family history of CVD was determined by answering (yes/no) if a first or second degree relative had a cardiovascular event at an early age (i.e. males < 55 years; females < 65 years). Questions pertaining to parental age, body mass, height, smoking status, and PA levels were also included. Parental PA levels were determined by asking how many times per week the parent engaged in any type of moderate PA for a minimum of 30 and 60 min, and how many times per week he/she was engaged in any type of vigorous PA for a minimum of 30 and 60 min. Moderate intensity was defined as being greater than resting, but less than somewhat hard, and being able to carry on a conversation when doing an activity. Vigorous intensity PA was indicated as “rapid heartbeat,” “hard breathing,” and/or sweating. PA levels were analyzed as continuous variables (i.e. frequency of moderate/vigorous physical activity in the past 7 days). Categories were also created to designate individuals who were sedentary (i.e. < 5 days/week of moderate PA; or <3 days/week of vigorous PA), or as habitually active (i.e. ≥ 5 days/week of moderate PA; or ≥3 days/week of vigorous PA).

### Statistical analysis

All statistical analyses were performed using SAS software version 9.1 (SAS Institute, Cary, NC). Normal distribution of each MetS component measurement was checked using a Shapiro-Wilks test in combination with graphical methods. Differences between the means of the sexes were determined by an independent-sample *t-test*, and sex difference in proportions was examined by Chi-square test. Bivariate analyses between all the variables under study were tested using a Pearson’s correlation. Gender-stratified multiple regression analyses were conducted to test the independent associations of each individual MetS risk factors and potential predictors. Explanatory variables included the variables reflecting individual lifestyles (i.e. CRF and PA), parents’ lifestyle indicators (i.e. smoking status and PA), and inheritable influence indicators (i.e. family history, parental BMI). Dummy coding was applied to categorical variables such as parental smoking status (yes = 1, no = 0) and family history of early CVD (yes = 1, no = 0). Linearity between dependent variable (i.e. waist circumference, SBP, DBP, glucose, log-transformed triglycerides and HDL) and the explanatory variables were checked graphically. Normality of the residuals was tested using a Shapiro-Wilks test and homogeneity of the variance of the residuals was tested by White’s General Specification test. Multicollinearity was tested using a variance inflation factor (VIF).

A continuous MetS risk score (MetScore) was created using PCA to determine the degree of clustering. The association between MetScore and various explanatory variables were analyzed using multiple regression analyses. In each model, MetScore was entered as the continuous dependent variable, and children’s PA and CRF were entered as independent predictors, along with potential mediating factors (i.e. parental smoking, BMI, and PA). Moreover, to determine the extent to which children’s BMI influences the association between PA, CRF and MetScore, we ran separate regression models with and without BMI. The PCA analysis revealed two principal components with eigen values ≥1.0 in both girls and boys. The first principal component (PC1) was correlated with all MetS components except glucose. The second principal component (PC2) was correlated with glucose, SBP and HDL. PC1 and PC2 accounted for 57% and 60% of MetS variance in females and males respectively.

## Results

Descriptive characteristics are presented in Table 1. Approximately 19% of children were overweight and 24% were obese. Female participants were younger, had smaller WCs, higher triglyceride levels, lower fasting glucose levels, lower CRF (estimated VO_2_), and were less likely to be physically active (see Additional file 2 and Additional file 3: Table S2).

**Table 1:** Characteristics of participants by sex

	**Girls**		**Boys**		**p**
	**n**	**Mean/Frequency**	**n**	**Mean/Frequency**	
**Children’s Factors**					
Age (yrs)	1486	11.5 ± 0.5	1390	11.6 ± 0.6	<.0001
BMI (kg/m^2^)	1486	21.4 ± 4.8	1390	21.2 ± 4.6	ns
Waist circumference (cm)	1478	70.9 ± 12.0	1378	73.9 ± 12.9	<.0001
SBP (mmHg)	1486	106.3 ± 10.4	1390	106.9 ± 10.7	ns
DBP (mmHg)	1486	68.9 ± 9.0	1390	68.8 ± 9.0	ns
HDL (mg/dl)	1486	51.1 ± 14.3	1390	51.7 ± 15.4	ns
Triglycerides (mg/dl)	1486	96.4 ± 57.8	1390	85.6 ± 48.2	<.0001
Glucose (mg/dl)	1486	93.2 ± 9.7	1390	94.8 ± 9.6	<.0001
VO_2_ (ml/kg/min)	1449	37.4 ± 4.2	1358	44.0 ± 3.7	<.0001
Physically active (%) ^a^	1406	25.3	1321	35.1	<.0001
**Parental Factors**					
Mother’s age (yrs)	1412	38.5 ± 5.6	1322	39.0 ± 5.5	0.02
Mother’s BMI (kg/m^2^)	1211	27.0 ± 6.1	1122	26.9 ± 6.3	ns
Mother Physically Active ^b^	1486	33.2	1390	37.0	0.03
Father’s age (yrs)	1339	40.8 ± 6.1	1261	41.1 ± 6.2	ns
Father’s BMI (kg/m^2^)	1149	28.9 ± 5.4	1058	28.8 ± 5.5	ns
Father Physically Active ^b^	1486	36.3	1390	37.2	ns
Family history% ^c^	1486	29.4	1390	30.2	ns
Mother smoke^d^	1486	28.3	1390	24.6	0.03
Father smoke^d^	1486	27.3	1390	27.2	ns

Abbreviations: *SBP* systolic blood pressure, *DBP* diastolic blood pressure, *BMI* body mass index calculated as body mass (kg) divided by height square (m2).

^a^ % of students who had moderate/vigorous physical activity for ≥1 h for ≥5 days per week.

^b^ % of students whose mother/father is physically active (defined as having ≥30 min of physical activity of moderate intensity ≥5 times/week or vigorous intensity ≥3 times/week).

^c^ % of students whose parents or grandparents had history of early onset cardiovascular diseases (diagnosed before 55 years of age for males and 65 for females).

^d^ % of children whose mother or father smokes.

### Independent predictors of single MetS risk components

Since each risk factor was correlated with at least one MetS component among girls and/or boys (see Additional files 4 and 5: Tables S3 and S4), they were used in a multiple regression model to test the independent association with each individual component. All significant partial regression coefficients and 95% confidence intervals (CIs) are provided in Table 2.

**Table 2:** Partial regression coefficient of independent predictors of MetS risks

**MetS Risk Components**	**Waist Circumference**	**Systolic Blood Pressure**	**Diastolic Blood Pressure**	**Fasting Glucose**	**HDL**	**Trig (Log)**
**Risk Factors: Girls**						
Children’s Factors						
BMI	1.94 (1.82-2.04)	0.68 (0.52-0.85)	0.33 (0.17-0.48)	-	−0.65 (−0.88 - -0.42)	0.03 (0.03 - 0.04)
CRF	−0.30 (−0.43 - -0.19)	−0.24 (−0.43 - −0.06)	-	-	- 0.50 (0.23 - 0.76)	-
PA	-	-	-	0.30 (0.08 - 0.52)	0.52 (0.11 - 0.93)	-
Parental Factors						
Mean Age	-	0.20 (0.09 - 0.32)	0.15 (0.04 - 0.25)	-	-	-
Mothers’ CVD History	-	-	-	-	−3.02 (−5.25 - −078)	-
Fathers’ CVD History	-	2.34 (0.79 - 3.89)	-	-	-	-
Mothers’ Smoking Status	-	-	-	1.41 (0.25 - 2.56)	-	-
Fathers’ BMI	-	-	0.13 (0.01 - 0.24)	-	-	-
**Risk Factors: Boys**						
Children’s Factors						
BMI	2.2 (2.08 - 2.32)	0.80 (0.59 - 1.01)	0.32 (0.15 - 0.50)	0.18 (0.03 - 0.33)	−0.53 (−0.84 - −0.22)	0.03 (0.02 - 0.03)
CRF	−0.39 (−0.54 - −0.24)	-	-	-	0.47 (0.07 - 0.87)	−0.02 (−0.03 - −0.004)
PA	−0.02 (−0.39 - − 0.04)	-	-	-	-	-
Parental Factors						
Mean Age	-	-	-	-	-	-
Mothers’ CVD History	-	-	-	1.4 (0.09 - 2.71)	-	-
Fathers’ CVD History	-	-	-	-	2.97 (−5.51 - −0.44)	-
Mothers’ Smoking Status	-	-	-	-	−3.22 (−5.72 - −0.72)	-
Fathers’ BMI	-	-	-	-	-	-

Significant beta values (95% confidence interval) are presented.

### Principal components analysis - MetScore derivation

The MetScore was computed by summing scores for PC1 and PC2, and each score was weighted for the relative contribution to the explained variance. The resulting MetScore was 0 ± 2.43 for girls and 0 ± 2.59 for boys.

### Independent predictors of the MetScore

Since all risk factors were correlated with at least one MetS component in girls and/or boys, they were all included in a multiple regression model to test the independent association with the MetScore (Table 3). For both girls and boys, BMI was independently associated with an increase in the MetScore, whereas CRF (estimated VO_2_) was negatively associated with it. Among girls, parental age and paternal family history of CVD were also associated with an increase in risk score; whereas in boys, maternal smoking status was associated with an increase in the MetScore. Without including children’s BMI in the model, maternal and paternal BMI and maternal smoking status were significantly associated with girls’ risk score. Among boys, the strong association between maternal smoking status and MetScore was not influenced by inclusion/exclusion of BMI in the model.

**Table 3:** Multiple regression models for independent predictors of PCA-derived MetScore, for both girls and boys

	**Model Predictor(s)**	**β**	**SE**	**t**	**Pr > |t|**	**95% CI**	**Adjusted R**^**2**^
PCA-derived MetScore							
Girls	Age	0.09	0.10	0.87	0.39	−0.11 - 0.29	0.52
	BMI	0.31	0.02	20.69	<0.01	0.28 - 0.34	
	CRF (VO2)	−0.07	0.02	−4.27	<0.01	−0.11 - −0.04	
	PA	0.00	0.03	0.02	0.99	−0.05 - 0.05	
	Mother’s BMI	0.00	0.01	0.08	0.94	−0.02 - 0.02	
	Mother’s CVD History	0.20	0.14	1.38	0.17	−0.08 - 0.48	
	Mother’s Smoking Status	0.26	0.14	1.88	0.06	−0.01 - 0.53	
	Mother’s PA	−0.01	0.02	−0.41	0.68	−0.04 - 0.03	
	Parental Age	0.02	0.01	2.12	0.03	0.002 - 0.04	
	Father’s BMI	0.01	0.01	0.95	0.34	0.01 - 0.03	
	Father’s CVD History	0.46	0.14	3.25	<0.01	0.18 - 0.75	
	Father’s Smoking Status	−0.13	0.14	−0.99	0.32	−0.40 - 0.13	
	Father’s PA	0.01	0.01	0.54	0.59	−0.02 - 0.04	
Boys	Age	0.13	0.12	1.14	0.25	−0.09 - 0.36	0.47
	BMI	0.31	0.02	15.35	<0.01	0.27 - 0.35	
	CRF (VO2)	−0.11	0.03	−4.25	<0.01	−0.16 - -0.06	
	PA	−0.03	0.03	−1.10	0.27	−0.09 - 0.03	
	Mother’s BMI	−0.01	0.01	−0.69	0.49	−0.03 - 0.01	
	Mother’s CVD History	0.10	0.17	0.58	0.56	−0.23 - 0.43	
	Mother’s Smoking Status	0.32	0.16	1.98	0.04	0.003 - 0.64	
	Mother’s PA	−0.01	0.02	−0.67	0.50	−0.05 - 0.03	
	Parental Age	0.01	0.01	0.81	0.42	−0.01 - 0.03	
	Father’s BMI	−0.01	0.01	−1.08	0.28	−0.04 - 0.01	
	Father’s CVD History	0.14	0.16	0.85	0.39	−0.18 - 0.46	
	Father’s Smoking Status	−0.12	0.16	0.78	0.44	−0.43 - 0.18	
	Father’s PA	0.01	0.02	0.29	0.77	−0.03 - 0.04	

## Discussion

We used principal components analyses to conduct a comprehensive investigation of cardiometabolic risk in a large cohort of 6th grade boys and girls. Our results reveal that both BMI and CRF are the two most influential independent predictors of overall risk in boys and girls, even after controlling for PA, family history of CVD, and parental characteristics (e.g. BMI, smoking status, age, etc.). The finding that CRF is inversely associated with metabolic risk has been previously shown [24,25]; however, given that PA serves as a moderating variable, the extent to which CRF may impact metabolic risk is often debated. Nevertheless, our data substantiate the importance of CRF as an independent determinant of metabolic risk in children. Moreover, we also identified novel sex-specific biological, behavioral, and parental predictors of childhood cardiometabolic risk. Specifically, among girls parental age and paternal family history of CVD were each independently associated with an increased Metscore. For boys, maternal smoking was the only additional independent predictor of an increased MetScore. Certainly, future research is needed to provide added insight into the mechanisms behind sex differences in cardiometabolic risks and divergent associations with environmental, familial, and behavioral influences.

Our findings suggest a critical need for tailoring gender-specific approaches to combat the increased prevalence of pediatric obesity, in order to ameliorate the impending comorbidity burden into adulthood. Our data are aligned with a recent investigation which demonstrated the independent contribution of CRF to MetS diagnosis among European children [26]. According to those results, individuals categorized with MetS were reported to have approximately 20–25% lower CRF than children without. This is also consistent with results from the European Youth Heart Study [27] which revealed that even after adjusting for habitual PA, CRF was a robust independent predictor of cardiovascular disease risk. In a large U.S. cohort of children we now confirm the heightened importance of addressing CRF independent of PA and BMI. This is aligned with recent findings by Jago et al. [28], which revealed that in 6th graders both fatness and fitness were independently associated with cardiometabolic risk.

Sex-differences in obesity prevalence were also observed, such that a higher proportion of boys were categorized as obese compared to girls (26 vs. 22%). This finding has been observed in prior population surveys (i.e. NHANES data [29]), and suggests that males and females indeed follow a divergent sex-specific trajectory in the development of risk for CVD [30] and related comorbidities (e.g. non-alcoholic fatty liver disease [31]). Although at present there is a lack of cut-points for WC in pediatrics (thus forcing the use of sex- and age-adjusted BMI cut points), abdominal obesity has been indicated as a primary driver of cardiometabolic risk [32]. In the current cluster analysis, WC was associated with all cardiometabolic risk factors and carried the strongest loading coefficient within the continuous MetScore outcome. Previous work has demonstrated that WC in children is the best predictor of adulthood MetS [33], and thus our findings bolster the importance of monitoring abdominal adiposity early and throughout pediatric clinical care.

**Figure 1:**
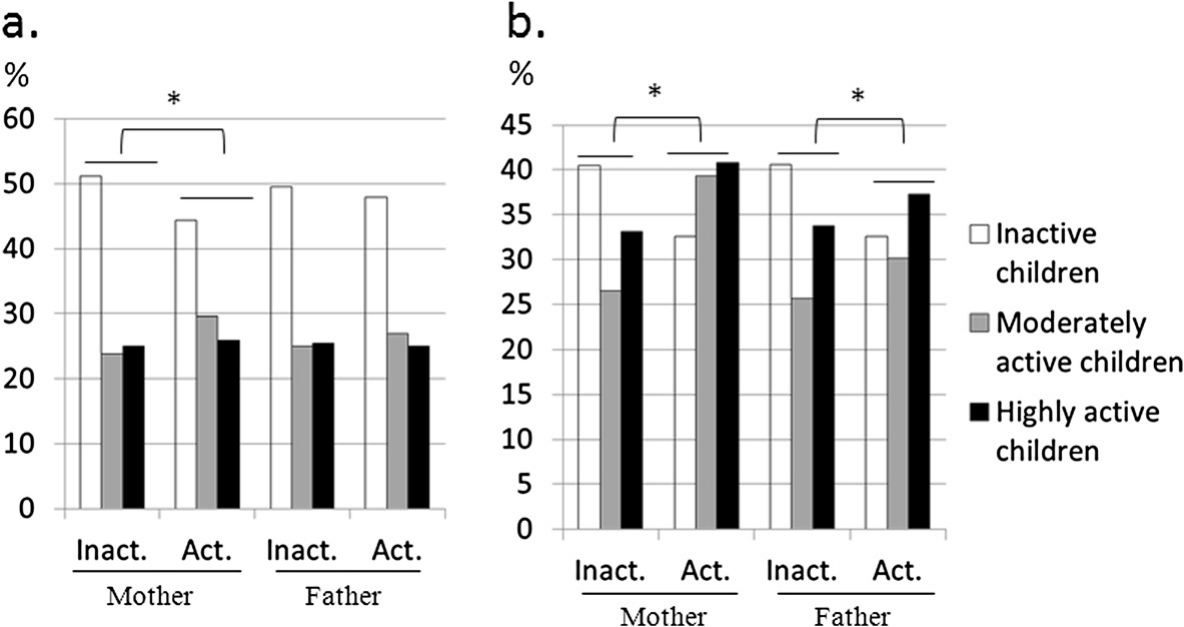
**Children’s physical activity status by Mother/Father’s physical activity status: a.) girls; and b.) boys.** Abbreviations/Notations: Inact, Inactive; Act, active; *, significant at p < 0.05 from Chi-Square test.

Cardiometabolic risk factors are known to aggregate in the family, which may be attributable to both hereditary and environmental influences [34,35]. In addition to a highly correlated BMI among parents and children, mothers who were physically active were more likely to have active children, as compared to those with inactive mothers (Figure 1). This was not the case for father’s activity level. Nevertheless, this is consistent with previous data indicating “familial clustering” in PA patterns [36], and suggests that optimizing children’s health is best achieved through comprehensive behavioral programs that involve the family-unit, and especially parental role modeling (particularly in mothers) through early adolescence.

**Figure 2:**
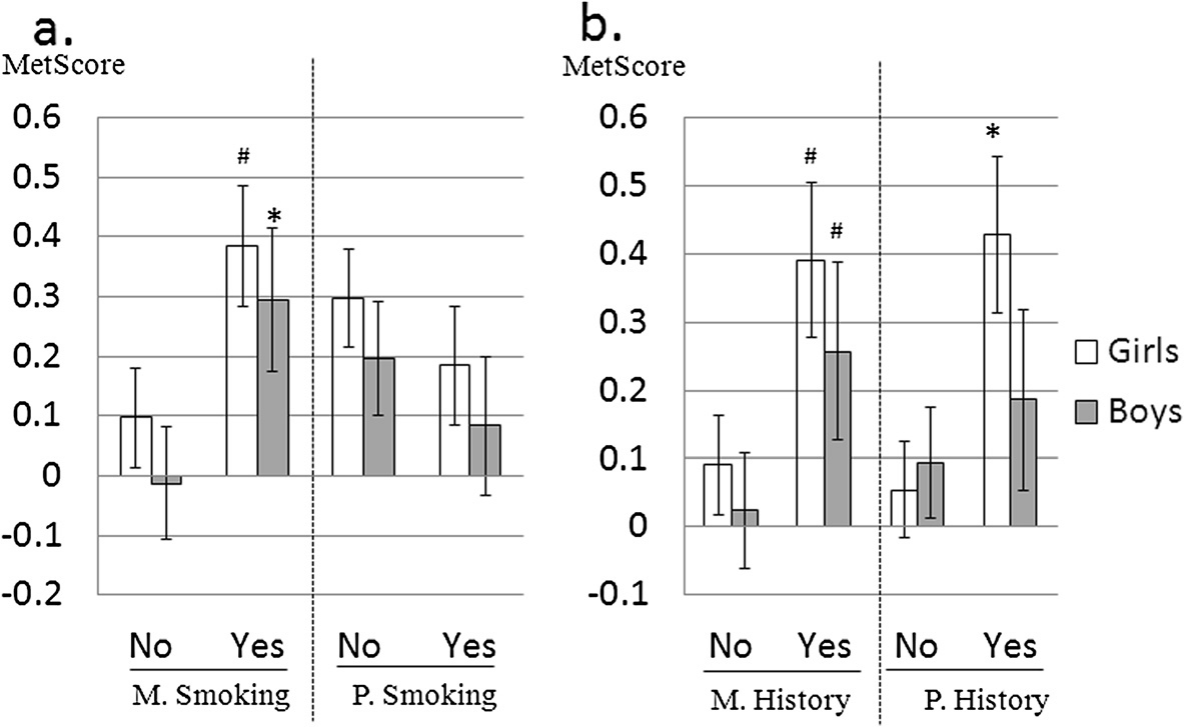
**Children’s MetScore by maternal/paternal smoking status and family history of early CVD.** MetScore mean was adjusted for children’s BMI, VO_2_ and Physical Activity levels. *, p < 0.05; #, p < 0.10 for the within gender difference between groups with (YES) and without (No) maternal/paternal smoking (panel a) and family history (panel b). M., maternal; P., Paternal.

Although maternal and paternal smoking status were significantly correlated with each other in our study, only maternal smoking was associated with MetScore in boys, and demonstrated a strong linear trend among girls (p = 0.06) (Figure 2). The adverse impact of parental smoking on children’s cardiovascular risk has been well established [37], particularly that of maternal smoking during pregnancy [38]. Nevertheless, our data extend these previous findings, by revealing the negative influence of current maternal smoking status on children’s cardiometabolic health. It is possible that current maternal smoking status may be reflective of an increased level of secondhand exposure among children. Environmental tobacco smoke exposure has been demonstrated to be independently associated with the MetS in adolescents [39], and it could be speculated that second hand exposure is substantially higher around mothers given that they are frequently the primary caregiver. On the contrary, it may also suggest a longstanding history of smoking, including smoking during pregnancy. Future research is certainly warranted to unravel the mechanistic influences of maternal smoking and second hand/environmental exposure on children’s cardiometabolic health risk.

Despite the clear connection between environmental influences and children’s cardiometabolic health, we cannot ignore the impact of non-modifiable heritable attributes when considering MetS risks [40]. In the current analyses parental family history of early CVD was independently associated with multiple individual MetS risk factors among girls and boys. Family history of early CVD has long been recognized as a risk factor in offspring [41], although evidence comparing the differential influence of maternal versus paternal family history on children’s risk for disease has been controversial. The “fetal origin hypothesis of adult chronic disease” [42] implies that stronger maternal-offspring associations for CVD risks are expected than for paternal-offspring associations. Conversely, our results suggest that paternal family history confers greater risk to girl’s cardiometabolic health than maternal family history, as this was the only non-modifiable familial factor associated with MetScore. This observation is consistent with a Framingham Heart Study [43], which revealed that paternal premature CVD was more robustly associated with coronary artery and abdominal aortic calcification among third generation young adults, as compared to maternal premature CVD.

### Study limitations

Caution is warranted when trying to generalize these findings to other ages and race/ethnic groups. Indeed, it is possible that unique biological, behavioral, and familial risk factors may exist for different races and ethnicities, and this should be a focus for subsequent investigations. We were unable to assess children’s pubertal stage and family socioeconomic status (SES). Tanner staging was not practical given our data collection procedures, and although all children were of similar age (i.e. in 6th grade), there may have been slight developmental differences within each gender which may account for minimal variability in cardiometabolic risk. Also, considering that boys and girls follow different developmental trajectories, we used a large gender equivalent population and stratified our analyses to focus on sex-specific risk, which limits the potential impact of pubertal development. Moreover, although individual SES was not obtained, mid-Michigan county-wide assessments using census tracking data revealed that children were generally from middle-income families-the largest SES population in the United States. Despite these issues, a considerable strength of this investigation was the homogeneity of the large study population, which reduced the potential confounding influence of disparities.

## Conclusions

The current findings using a robust analytic approach revealed numerous modifiable biological and behavioral factors that shared strong associations with boys’ and girls’ cardiometabolic health risk. Accordingly, both BMI and CRF represent ideal targets for prevention/treatment therapies in boys and girls, to reduce the cardiometabolic health burden. Moreover, the novel sex-specific risk factors identified such as parental age, paternal family history of CVD, and maternal smoking, also influence metabolic risk in girls and boys, respectively. Additional exploration of these factors and other fitness attributes (e.g. muscular strength [44]) may lead to a better understanding of family-based and gender-specific cardiometabolic risk in childhood, and sustainable approaches for chronic disease prevention.

## Abbreviations

BMI: Body Mass Index; CDC: Centers of Disease Control; CRF: Cardiorespiratory fitness; CVD: Cardiovascular disease; DBP: Diastolic blood pressure; HDL-cholesterol: High-density lipoprotein – cholesterol; LDL-cholesterol: Low-density lipoprotein – cholesterol; MetS: Metabolic syndrome; MetScore: Metabolic syndrome score; NHANES: National Health and Nutrition Examination Survey; PA: Physical activity; PCA: Principal components analysis; SBP: Systolic blood pressure; TC: Total cholesterol; TG: Triglycerides; VIF: Variance inflation factor; VO_2_: Volume of oxygen consumption; WC: Waist circumference; YRBSS: Youth Risk Behavior Surveillance System.

## Competing interests

The authors declare that they have no competing interests.

## Authors’ contributions

PG, PV, and WS designed the study and carried out data collection. MP and DL conducted the statistical analysis. MP, DL, HI and PG participated in writing and editing the manuscript. All authors read and approved the final manuscript.

## Supplementary Material

Additional file 1 **Table S1.** Pairwise correlations for MetS risk factors. Click here for file

Additional file 2 Text description of bivariate correlates for MetS risk. Click here for file

Additional file 3 **Table S2.** Bivariate associations between MetS risk factors and potential correlates of individual and familial factors. Click here for file

Additional file 4 **Table S3.** Bivariate correlations between explanatory variables in girls. Click here for file

Additional file 5 **Table S4.** Bivariate correlations between explanatory variables in boys. Click here for file
