# Supplementary material for: Principal component analysis reveals gender-specific predictors of cardiometabolic risk in 6th graders
Source: Cardiovasc Diabetol. 2012 Nov 28;11:146. doi: 10.1186/1475-2840-11-146 (PMC3537600; doi:10.1186/1475-2840-11-146)
Supplement: Additional file 1 — Table S1. Pairwise correlations for MetS risk factors. [file 1475-2840-11-146-S1.docx]

**Supplemental File 1: Pairwise correlations for MetS risk factors**

| Girls | | | | | |
| --- | --- | --- | --- | --- | --- |
|  | SBP | DBP | HDL | LogTG | Glucose |
| WC | 0.38* | 0.25* | -0.28* | 0.36* | 0.07* |
| SBP |  | 0.55* | -0.09* | 0.18* | 0.09* |
| DBP |  |  | -0.08* | 0.11* | 0.04 |
| HDL |  |  |  | -0.35* | 0.12* |
| Trig (log) |  |  |  |  | 0.03 |
| Boys | | | | | |
| WC | 0.36* | 0.23* | -0.26* | 0.37* | 0.08* |
| SBP |  | 0.49* | -0.10* | 0.18* | 0.08* |
| DBP |  |  | -0.09* | 0.14* | 0.04 |
| HDL |  |  |  | -0.30* | 0.04 |
| Trig (log) |  |  |  |  | 0.13* |
