# Supplementary material for: Principal component analysis reveals gender-specific predictors of cardiometabolic risk in 6th graders
Source: Cardiovasc Diabetol. 2012 Nov 28;11:146. doi: 10.1186/1475-2840-11-146 (PMC3537600; doi:10.1186/1475-2840-11-146)
Supplement: Additional file 2 — Text description of bivariate correlates for MetS risk. [file 1475-2840-11-146-S2.docx]

**Supplemental File 2:**

**Bivariate Correlates of MetS Risk.** Bivariate correlation analyses (**Supplemental Files 1 and 3**) indicated that most MetS components were significantly intercorrelated, and moreover, that age, BMI, estimated VO_2_, physical activity, maternal BMI, paternal BMI, and maternal smoking status were each significant correlates to at least three cardiometabolic risk factors. Further, maternal age, family history of CVD, and paternal family history were also correlated with 1-2 risk factors in girls and boys. Parental PA levels were correlated with boys’ blood pressure. Smoking status of the father was significantly correlated with girls’ WC and boys’ fasting glucose levels. Since maternal and paternal age were highly correlated (r>0.70, p<.0001), and in order to minimize issues of multicollinearity, parental age was calculated as the mean of maternal and paternal age for use in the regression analysis.
