# Supplementary material for: Principal component analysis reveals gender-specific predictors of cardiometabolic risk in 6th graders
Source: Cardiovasc Diabetol. 2012 Nov 28;11:146. doi: 10.1186/1475-2840-11-146 (PMC3537600; doi:10.1186/1475-2840-11-146)
Supplement: Additional file 3 — Table S2. Bivariate associations between MetS risk factors and potential correlates of individual and familial factors. [file 1475-2840-11-146-S3.docx]

**Supplemental File 3: Bivariate associations between MetS risk factors and potential correlates of individual and familial factors.**
