# Supplementary material for: Principal component analysis reveals gender-specific predictors of cardiometabolic risk in 6th graders
Source: Cardiovasc Diabetol. 2012 Nov 28;11:146. doi: 10.1186/1475-2840-11-146 (PMC3537600; doi:10.1186/1475-2840-11-146)
Supplement: Additional file 4 — Table S3. Bivariate correlations between explanatory variables in girls. [file 1475-2840-11-146-S4.docx]

**SDC 4: Bivariate correlations between explanatory variables in girls.**
